# The autophagy research in electron microscopy

**DOI:** 10.1186/s42649-019-0012-6

**Published:** 2019-11-06

**Authors:** Minkyo Jung, Hyosun Choi, Ji Young Mun

**Affiliations:** 1 Neural circuit research group, Korea Brain Research Institute, Daegu, Korea; 2 BK21 Plus Program, Department of Senior Healthcare, Graduate School, Eulji University, Daejeon, Korea

**Keywords:** Electron microscopy, Autophagic flux, CLEM, Immuno-gold, Cryo-EM

## Abstract

Autophagy, a highly conserved process of eukaryotic cellular recycling, plays an important role in cell survival and maintenance. Dysfunctional autophagy contributes to the pathologies of many human diseases. Many studies have attempted to clarify the process of autophagy. Here, we review morphological studies of autophagy involving electron microscopy.

## Introduction

In the late 1950s, electron microscopy (EM) studies identified the autophagosome as a mitochondria surrounded by a larger vesicular structure (Rhodin 1954). Since this discovery, EM has been the main tool used to study autophagy (Rhyu 2017). Autophagy is a well-known lysosomal degradation pathway and a major factor in cellular clearance mechanisms. The ubiquitin–proteasome system degrades short-lived or abnormally folded proteins, while the lysosome-autophagy process targets long-lived macromolecular complexes and organelles. Defects in autophagy decrease the removal of potential sources of genotoxic stress, such as reactive oxygen species (ROS), from damaged and leaky mitochondria or other organelles (White 2012). Accumulated ROS may be an important cause of DNA damage and genetic instability. Neurodegenerative disease is a main consequence of autophagic failure. The definition of autophagy covers three general types of mechanisms, depending on the pathway used to deliver cargo: macroautophagy, microautophagy, and chaperone-mediated autophagy (CMA). Macroautophagy and microautophagy are conserved in all eukaryotes, whereas CMA seems to be specific to higher eukaryotes. In macroautophagy, a double- or multi-membraned autophagosome fuses with a lysosome to enable nonspecific degradation. In contrast, microautophagy involves direct engulfment by the lysosome. Although these two systems were visualized in an EM image of the rat liver in 1966 (De Duve and Wattiaux 1966), microautophagy was not as well clarified. In this review, we describe several techniques used to understand various autophagic processes.

## Main text

### Electron microscopy with conventional fixation and plastic sectioning

**Fig. 1:**
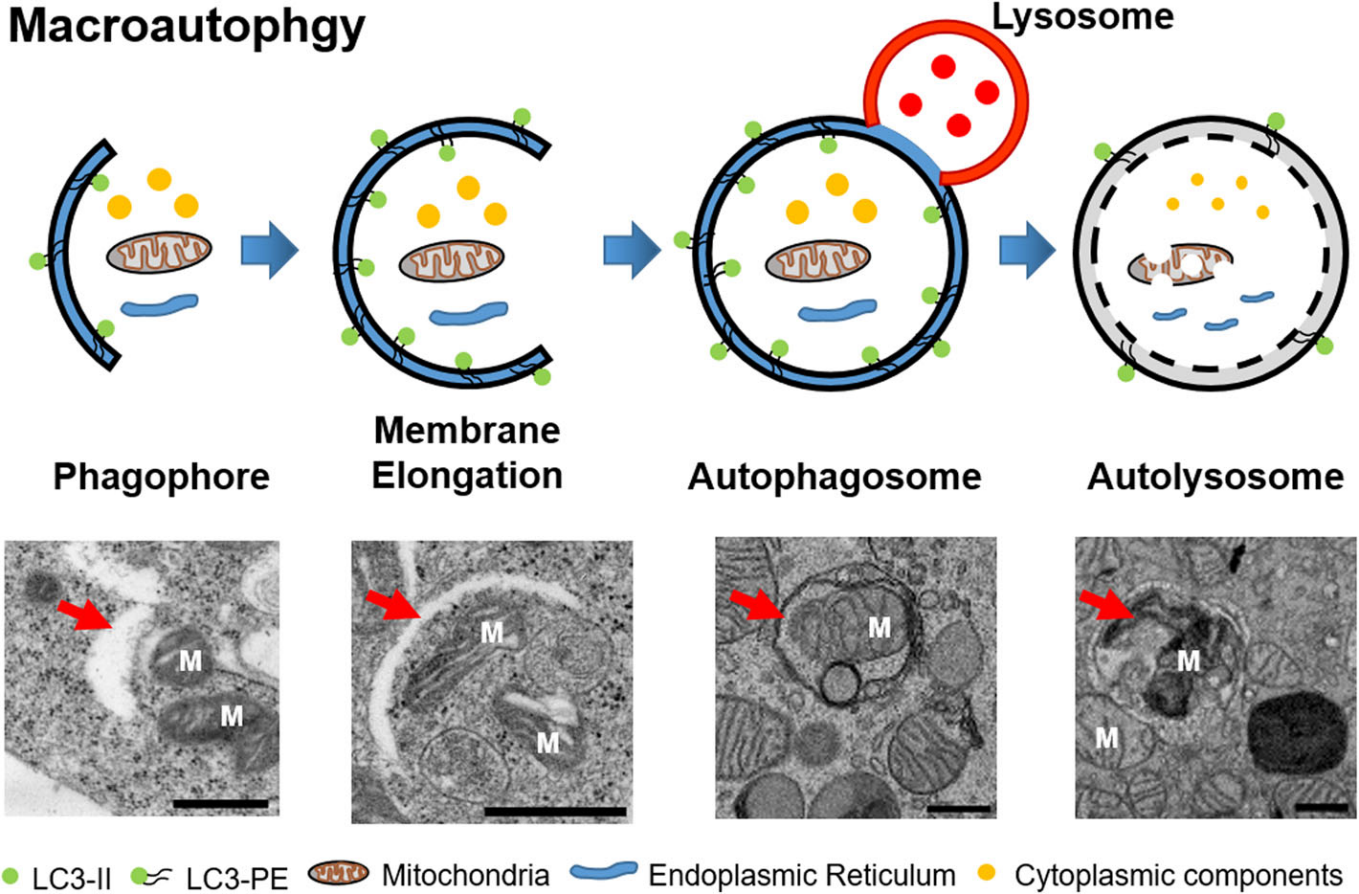
Schematic model and transmission electron microscopic image of macroautophagy. Autophagy is a multi-step process including four main processes, which is isolation of membrane, vesicle expansion, maturation and lysosome fusion, and degradation of compartments. M: mitochondria, scale bar: 500 nm

Protocols for the preparation of samples (Arai and Waguri 2019; Swanlund et al. 2010; Yla-Anttila et al. 2009b) have been developed for transmission electron microscopy (TEM)-based analyses of autophagy. Conventional fixation methods using glutaraldehyde, paraformaldehyde and osmium tetroxide have been used in studies of autophagy. Notably, the imidazole-buffered osmium tetroxide protocol presents unsaturated lipids, including the limiting membranes of autophagosomes, in high contrast, which facilitates their identification even at low magnification. After conventional sample preparation, ultrathin (70–80 nm) sections of cells are cut from a plastic block and used to observe autophagy. This method has been used for autophagy research for more than 60 years. Researchers have reported changes in autophagy flux according to the numbers of early-stage autophagosomes and late-stage autophagic vacuoles (Cheng et al. 2015; D'Assante et al. 2017; Martin et al. 2011). Macroautophagy is characterized by unique morphological features, including the sequestering vesicles known as autophagosomes which differ from other vesicles that bud from preexisting organelles (Fig. 1). Although autophagy is initiated, normal autolysosome’s formation cannot be followed. EM can distinguish the specific morphology and abnormal morphological features, such as incomplete fusion of the autophagosome and lysosome (Bustos et al. 2017), depending on the condition of the cell. Regarding microautophagy, Glaumann and colleagues demonstrated a flab or arm-like protrusion on an isolated rat liver lysosomal membrane that enabled the engulfment of particles (de Waal et al. 1986; Marzella et al. 1980). Although the term microautophagy was first used in 1966 by De Duve and Wattiaux (De Duve and Wattiaux 1966), little was known about the mechanism. Conventional EM has led to the description of different forms of autophagy as mitophagy (Chen et al. 2017), lipophagy (Tarique et al. 2019) and pexophagy (Schrader and Fahimi 2008), depending on the cargo inside the autophagosome. In case EM can depict autophagosomes containing mitochondria, lipids, or peroxisomes, these structures can be distinguished. However, it can be difficult to depict clearly the sizes and total volumes of the different compartments inside the cells using this conventional method because thin sections of cells are analyzed instead of whole cells. The embedding of flat adherent cultured cells, rather than a cell pellet, facilitated an understanding of autophagy flux in whole cells (Yla-Anttila et al. 2009b). However, the total volumes and accurate sizes of specific structures could not be understood in a single plane of a sectioned image. A three-dimensional (3D) electron tomography structure based on a 250-nm thick section revealed connections between detail structures of the phagophore and endoplasmic reticulum (Yla-Anttila et al. 2009a). Yla-Anttila and colleagues used electron tomography to depict a cup-shaped membranous structure, known as a phagophore, and a mature autophagosome. In their TEM analysis of cultured cells, the autophagosomal diameter varied between 300 nm and several micrometers, with an average of 600 nm. The 3D structure revealed details of autophagic process. However, it was not sufficient to depict specific details of the autophagic process because of the requirement for protein labeling.

### Correlative light and electron microscopy

**Fig. 2:**
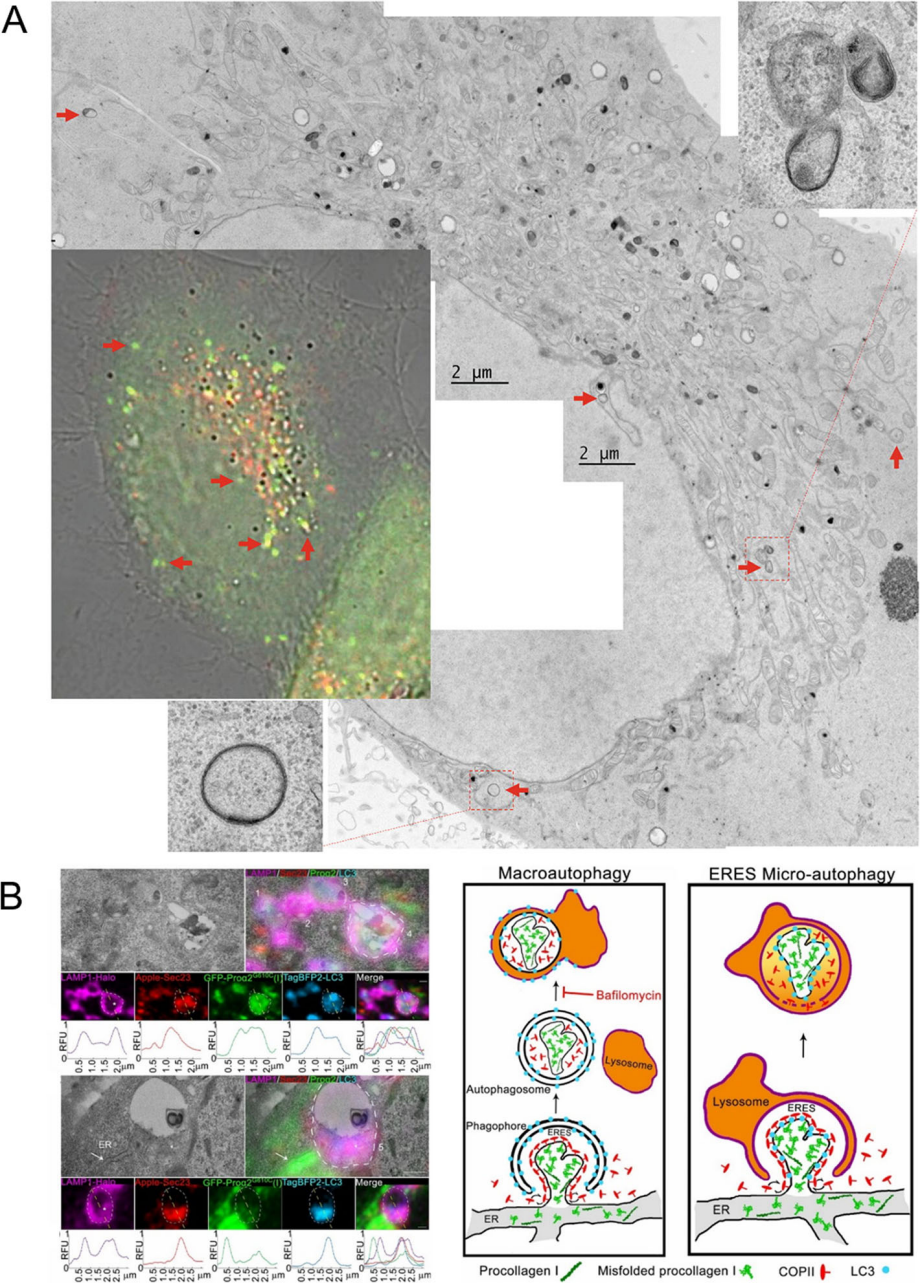
Example of correlative light and electron microscopy of autophagosomes. **a** The red arrow indicates the corresponding structure between the fluorescence image and the electron microscope image. The higher magnification inserted images show that the morphology of the autophagosomes. **b** Ultrastructure and correlative image of ERESs engulfed by lysosomes and proposed noncanonical ERES microautophagy model. Reproduced with permission from the Springer Nature (Gudmundsson et al. 2019) and Proceedings of the National Academy of Sciences (Omari et al. 2018)

Correlative light and EM (CLEM) combines light microscopy (LM) with high-resolution EM. The combination of the various labels used in LM imaging with the nanoscale high-resolution structure provided by EM makes correlative microscopy superior for morphological studies of different types of autophagy. The canonical formation of an autophagosome involves four steps: initiation, nucleation, elongation, and closure. However, another type of autophagy, noncanonical autophagy, has been described. Experimental characterizations of noncanonical autophagy have reported that double-membraned autophagosomes do not elongate from a single source and are not necessary for the intervention of ATG proteins. Omari and colleagues used CLEM to visualize noncanonical autophagy, which occurs at endoplasmic reticulum (ER) exit sites (ERESs) through a microautophagy-like mechanism. These authors used live-cell confocal microscopy and CLEM to investigate the recognition and capture of misfolded procollagen for autophagic degradation (Omari et al. 2018). Omari and colleagues used LC3, Sec23, and LAMP1 to depict the ultrastructures of ERESs engulfed by lysosomes on CLEM images. These internal lysosomal membrane-containing structures suggested microautophagy rather than macroautophagy, and this process was unique as it lacked a double-membrane autophagosome. Recently, the Eskelinen group introduced CLEM technique for live-cell imaging that used fluorescently tagged LC3 to study autophagosome biogenesis and maturation (Gudmundsson et al. 2019) (Fig. 2). However, studies that label different stages of autophagy are needed to reveal more details about the autophagic progress.

### Immuno-gold labeling of proteins in autophagy flux

**Fig. 3:**
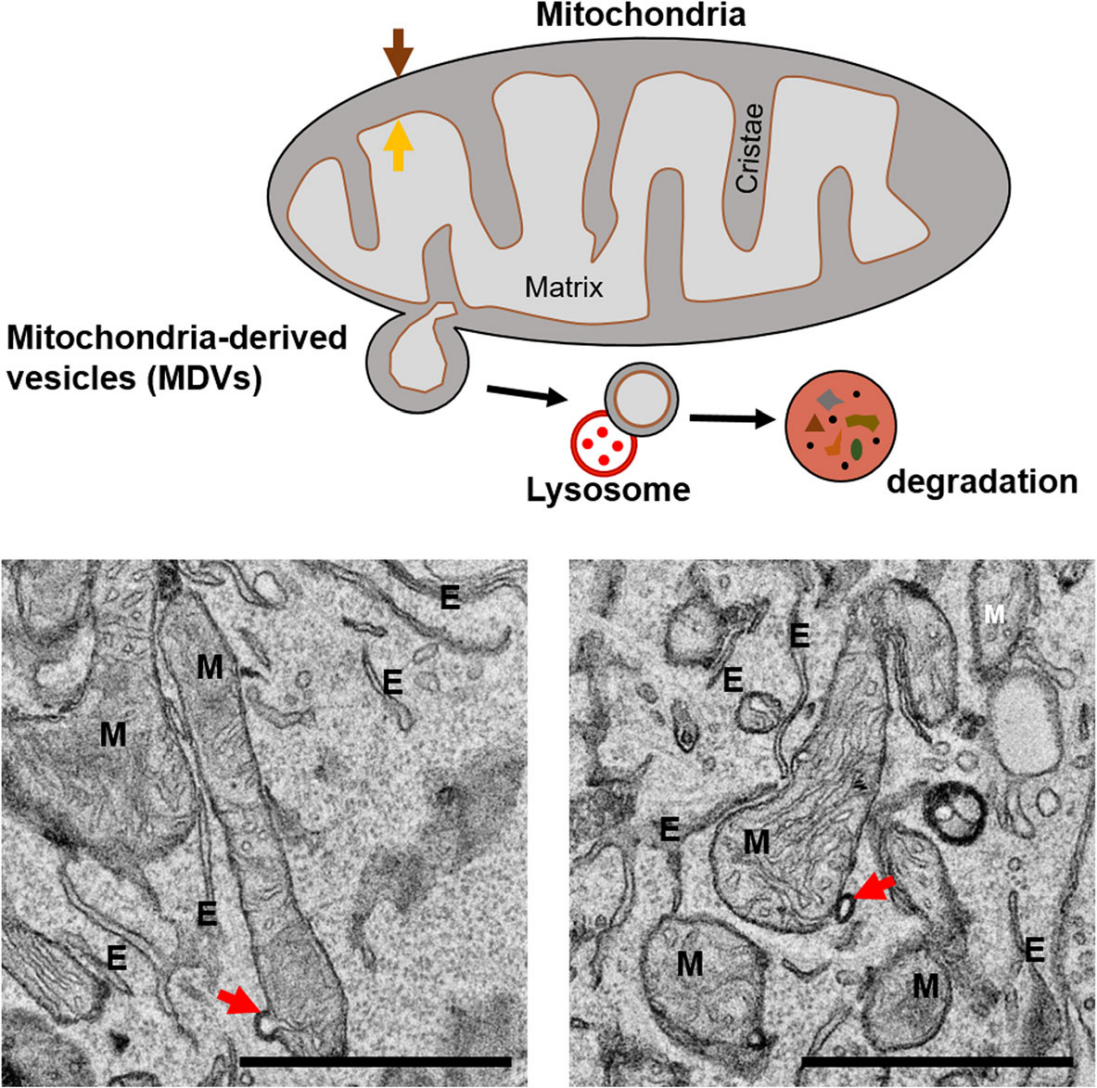
Schematic model and electron microscopic image of mitochondrial-derived vesicles (MDVs). MDVs are generated from the outer mitochondria membrane and include outer, inner membrane, and matrix compartments (Red arrow). Brown arrow: outer membrane, Orange arrow: inner membrane, M: mitochondria, E: Endoplasmic reticulum, Scale bar: 1 μm

Immuno-gold labeling and EM have been used to study the functions of specific proteins in autophagy. Immuno-gold labeling, which is based on antigen–antibody reactions, is used to investigate the locations of target proteins. Hamasaki and co-workers used immunoelectron microscopy to observe that green fluorescent protein (GFP)-tagged ATG14, a pre-autophagosome/autophagosome marker, accumulated at the sites of contact between the ER and mitochondria under conditions of starvation via Tokuyasu sampling (Tokuyasu and Singer 1976). In addition, Hamasaki and colleagues demonstrated that the autophagosome formation marker ATG5 also localizes at the sites of ER–mitochondria contact until the autophagosome has formed completely (Hamasaki et al. 2013). The immuno-gold technique revealed that autophagosome biogenesis might initiate at the sites of ER–mitochondria contact, as well as the mitochondria themselves (Hailey et al. 2010) or ER (Yla-Anttila et al. 2009a). This technique has also been used to study the initiation of selective pathways of autophagy, such as mitophagy. Parkin and pink, which have been reported as factors involved in the initiation of mitophagy, were detected using immuno-gold labeling (Cook et al. 2014). By discovery through immuno-gold labeling, the distribution of parkin in mitochondria containing vacuoles than other mitochondria without vacuoles, the authors demonstrated that the autophagosome did not form around parkin-labeled mitochondria; rather, material from the mitochondrial membrane contributed to the formation of the developing autophagosome. Mitophagy is a specialized type of autophagy that regulates the turnover of damaged and dysfunctional mitochondria. The mitochondria are organelles that produce cellular energy in the form of ATP and regulate energy homeostasis. Mitophagy is important for maintaining a healthy and closely regulated mitochondrial population. Sugiura et al. reported the mitochondria-derived vesicle (MDV), a TOM20-immunopositive vesicle that budded from mitochondria, as a new short-term mitophagy pathway (Sugiura et al. 2014). Cadete and colleagues reported that MDVs develop readily in cardiac tissues in mice under normal, healthy conditions and suggested that MDV formation, rather than mitophagy, acts as a first line of defense against acute stress (Cadete et al. 2016). The schematic model and EM images were shown in Fig. 3. The subtypes of autophagic vacuoles (AVs) can also be classified using immuno-gold labeling. For example, immuno-gold labeling using antibodies specific for cathepsin D, an aspartic protease that localizes to the endosomes and lysosomes, detected more mature AVs. The authors demonstrated that many neurites in a human brain with Alzheimer’s disease contained high proportions of cathepsin D-negative (i.e., immature) AVs (Nixon et al. 2005), suggesting that the transport of AVs and their maturation to lysosomes may be impaired. In addition, immuno-gold labeling has been used widely to study the functions of autophagy using the locations of specific proteins.

### Negative staining and 2D averaging of protein complexes in autophagy

Negative staining is the most common EM technique used to determine protein structures (Scarff et al. 2018). This method enables the relatively simple and rapid observation of macromolecules and macromolecular complexes using a contrast-enhanced staining solution, such as uranyl acetate or uranyl formate. However, this technique has a limited resolution and is mainly used to assess samples before cryo-TEM. However, negative staining is a powerful EM technique for the structural studies of protein and protein complexes when combined with 2D class averaging. Negative stained EM was also used to visualize the core mechanistic factors of autophagy, such as the Atg17–Atg31–Atg29 complex. The S-shape of this complex suggested that it may contribute to high vesicular curvature. This S-shaped structure could be visualized using 2D classification (Chew et al. 2013; Mao et al. 2013), which is used to increase the signal-to-noise ratio and enhance finer details via image averaging (Ohi et al. 2004). The S-shaped Atg17–Atg31–Atg29 complex suggested PAS organization and autophagy induction. Chew and colleagues also used negative staining EM and 2D averaging to demonstrate that Atg17 mediates dimerization and conformational flexibility (Chew et al. 2013). Later, they reported that Atg13 serves as a bridge to the catalytic Atg1 subunit in the Atg17–Atg31–Atg29 complex (Chew et al. 2015). Negative stained EM, 2D averaging and 3D reconstruction were also used to reveal the V-shaped structure of the phosphatidylinositol 3-kinase complex 1 (PI3KC3–C1 complex), which is involved in autophagy initiation (Baskaran et al. 2014). This complex comprises the lipid kinase VPS34, scaffolding protein VPS15, tumor suppressor BECN1 and autophagy-specific subunit ATG14. The two arms of the V shape consist of the largest protein, VPS15, which acts as a bridge between BECN1 and VPS34. This connection forms a flexible structure with one inflexible arm, suggesting that this structural characteristic may be useful for drug design. A detailed higher-resolution structure was also studied using cryo-EM.

### Cryo-EM of protein related to autophagy

Cryo-EM provides higher-resolution images because proteins are not covered in a contrast-enhanced staining solution (Cressey and Callaway 2017). The Nobel prize winner Dubochet and colleagues developed a liquid ethane-based technique for the rapid freezing of proteins that prevents the dehydration of water-soluble biomolecules in the vacuum of an electron microscope. Subsequently, Henderson and Frank, also Nobel prize winners, developed software to reconstruct the Cryo-EM images into 3D structures. This software allowed researchers to use EM to determine the structures of proteins at much higher resolutions than were previously available, and it is considered as a resolution revolution (Kuhlbrandt 2014). The PI3KC3–C1 complex was studied using cryo-EM and a single particle analysis (Ma et al. 2017). Specifically, Ma et al. reported the cryo-EM structures of human PI3KC3–C1 and PI3KC3–C2 at a subnanometer resolution. These authors also visualized the orientations of the complexes on membranes by deleting ATG14L or the C terminus of VPS34. The study results demonstrated that the C terminus of ATG14L is responsible for anchoring C1 on membranes, while the C-terminal VPS34 deletion mutant determined the orientation of the complex. Autophagosomes are thought to emerge from omegasomes in the ER. One report suggested that omegasome formation may be related to the phosphorylation of ER via PI3KC3 kinases (Nascimbeni et al. 2017). Structural data based on cryo-TEM suggested the process by which the kinases were recruited to the ER. This process required ATG14L-BATs and mediated the binding of PI3KC3 to the membranes, suggesting that the complex interacts directly with the ER membrane. Purified proteins are useful for cryo-EM-based structural studies. However, more advanced EM techniques, such as cryo-electron tomography (cryo-ET) using cryo-sectioning (CEMOVIS), and new approaches involving combinations with the cryo-Focused Ion Beam (cryo-FIB) are available for studies of unknown autophagic processes within the cell.

## Conclusions

Autophagy was originally described using electron microscopy approximately 50 years ago, when electron microscopy and sample preparation methods for biological materials had just emerged. The field has expanded exponentially since the discovery of autophagy genes, and this biological process has increasingly gained attention. Despite major developments in various methods used to monitor autophagy in cells and organisms, EM provides necessary qualitative and quantitative information that cannot be obtained using other methods. In the field of EM, cryofixation and tomography are likely to provide high-resolution 3D images of autophagic compartments that are free of artifacts caused by chemical fixation. These characteristics are very likely to elucidate unanswered questions in the field of autophagy field. Higher-resolution imaging and specific labeling techniques based on advanced EM, including CLEM and CEMOVIS, are needed to investigate various unanswered questions regarding the mechanisms of autophagy regulation.

## Data Availability

The datasets used and/or analysed during the current study are available from the corresponding author on reasonable request.
